# Risk factors for the incursion, spread and persistence of the foot and mouth disease virus in Eastern Rwanda

**DOI:** 10.1186/s12917-020-02610-1

**Published:** 2020-10-12

**Authors:** Jean Claude Udahemuka, Gabriel Oluga Aboge, George Ogello Obiero, Phiyani Justice Lebea, Joshua Orungo Onono, Massimo Paone

**Affiliations:** 1 Centre for Biotechnology and Bioinformatics, University of Nairobi, P.O. Box 30197, Nairobi, Kenya; 2 Department of Veterinary Medicine, University of Rwanda, P.O. Box 57, Nyagatare, Rwanda; 3 Department of Public Health, Pharmacology and Toxicology, Faculty of Veterinary Medicine, University of Nairobi, P.O. Box 29053, Nairobi, Kenya; 4 TokaBio (Pty), Ltd, Pretoria, South Africa; 5 Food and Agriculture Organization of the United Nations, Viale delle Terme di Caracalla, Rome, Italy

**Keywords:** Foot-and-mouth disease, Risk factors, Maps, Geospatial, QGIS, Eastern Rwanda, Foot-and-mouth disease spread

## Abstract

**Background:**

Identification of risk factors is crucial in Foot-and-mouth disease (FMD) control especially in endemic countries. In Rwanda, almost all outbreaks of Foot-and-Mouth Disease Virus (FMDV) have started in Eastern Rwanda. Identifying the risk factors in this area will support government control efforts. This study was carried out to identify and map different risk factors for the incursion, spread and persistence of FMDV in Eastern Rwanda. Questionnaires were administered during farm visits to establish risk factors for FMD outbreaks. Descriptive statistical measures were determined and odds ratios were calculated to determine the effects of risk factors on the occurrence of FMD. Quantum Geographic Information System (QGIS) was used to produce thematic maps on the proportion of putative risk factors for FMD per village.

**Results:**

Based on farmers’ perceptions, 85.31% (with *p* < 0.01) experienced more outbreaks during the major dry season, a finding consistent with other reports in other parts of the world. Univariate analysis revealed that mixed farming (OR = 1.501, *p* = 0.163, CI = 95%), and natural breeding method (OR = 1.626; *p =* 0.21, CI = 95%) were associated with the occurrence of FMD indicating that the two risk factors could be responsible for FMD outbreaks in the farms. The occurrence of FMD in the farms was found to be significantly associated with lack of vaccination of calves younger than 12 months in herds (OR = 0.707; *p* = 0.046, CI = 95%).

**Conclusions:**

This is the first study to describe risk factors for persistence of FMDV in livestock systems in Rwanda. However, further studies are required to understand the role of transboundary animal movements and genotypic profiles of circulating FMDV in farming systems in Rwanda.

## Background

Foot-and-Mouth Disease (FMD) is a highly contagious viral disease caused by a picornavirus known as Foot-and-Mouth Disease Virus (FMDV) [1]. FMD affects cloven-hoofed animals including domestic and wild animals [2]. Cattle, sheep, goats and pigs are the most important domestic animals affected by the disease. In wildlife, at least 70 species of wild and captive animals including African buffaloes (*Syncerus caffer*) are affected [2]. The East African region is considered to have the most complicated situation with regard to the control of FMD. This is due to interactions between domestic and wild animals susceptible to FMD, uncontrolled transboundary animal movements and high genetic diversity of FMDV in the region [3]. The Akagera National Park (ANP) is home to many FMD susceptible wild animals, which are at of risk of interacting with livestock in farms adjacent to ANP. Rwanda has experienced many outbreaks of FMD with serotypes O reported in 1960, 1998, 2004 2008, 2009 and 2010 [4]. On the other hand, the first outbreak of SAT2 was reported in 1992 with the subsequent outbreaks being reported in 1996–1997, 2000–2001, 2004, 2005, 2006, 2008, 2009, 2010, 2013, 2015 and 2017 [4–9]. The other outbreaks of FMD involving serotypes A and SAT1 occurred in 2008, 2009, 2010 [9] and again serotype SAT1 in 2012–2013 [10].

Despite the reports of the previous outbreaks of the disease, little is known about risk factors responsible for these outbreaks. Understanding these risk factors is needed for the development of a Risk Assessment Plan necessary for the advancement in the Progressive Control Pathway for Foot-and-Mouth Disease (PCP-FMD) stages [11].

The Eastern Province of Rwanda is the largest province having 9813 km^2^ with a predominantly sedentary farming system. The livestock population in Eastern Province is composed of approximately 500,000 cattle, 500,000 goats, 13,000 sheep and 130,000 pigs [12]. This province neighbours Uganda and Tanzania and uncontrolled transboundary animal movements are likely to occur here. Moreover, the province receives low rainfall and is characterized by the absence of water bodies such as rivers. Consequently, many farmers in this province tend to use communal watering points thereby encouraging the congregation of animals and eventually contributing to the spread of FMD outbreaks [13]. In this regard, this area has been known to be a hotspot for most of the FMD outbreaks in Rwanda for the last two decades [12]. The dry season seems to be the period during which FMD outbreaks are more likely to occur in Eastern Rwanda and surrounding areas in Uganda and Tanzania [14–16]. During the dry season, there is a shortage of pasture and water; thereby forcing most livestock farmers to move their animals in search of pasture and water. This encourages contacts between infected and non-infected animals during an outbreak of FMD.

### Statement of the problem

Nevertheless, fewer studies have been done to identify and map the risk factors responsible for FMD outbreaks in East Africa classified as FMD pool 4 where serotypes O, A, SAT1, SAT2 and SAT3 have been isolated [17]. Some of the risk factors which includes dry season and animal movements have been documented in Uganda and Tanzania [18–20]. Other putative risk factors, such as the use of shared bulls, for small-scale dairy farmers have also been reported in Kenya [21]. Despite these few studies in the region, none has been reported in Eastern Rwanda. We conducted questionnaire-based surveys in Nyagatare and Gatsibo districts of the Eastern province in Rwanda, known to be a hotspot for FMD outbreaks in the country [22] to investigate FMD risk factors.

### Purpose and what was done

Comprehensive knowledge of risk factors is key in developing the Risk-Based Strategic Plan for the control of FMD required for achieving and maintaining the PCP-FMD level 2 [11]. Therefore, from March 2018 to June 2018 we conducted surveys to investigate risk factors responsible for the incursion, spread and persistence of FMD in Eastern Rwanda. In addition, we produced thematic maps for a spatial understanding of these risk factors.

## Results

We interviewed 184 farmers in 19 villages of Nyagatare and Gatsibo districts. However, considering that, some farmers did not answer all the required questions and hence unsuitable for analysis, we only exploited 143 responses. Among the 143 respondents, 36 (25.17%) of them reported having had at least one FMD outbreaks within the last 5 years in their farms. There was no active outbreak of FMD in the visited farms during the interviewing period, the last outbreaks had occurred between May 2017 and February 2018 and were caused by serotypes SAT 2 [7]. During the outbreak, local veterinary officers collected oropharyngeal fluids, tissue and blood samples. The samples were shipped to the Virology laboratory of the Rwanda Agriculture Board to confirm the outbreak by Enzyme-Linked Immunosorbent Assay (ELISA) and Polymerase Chain Reaction (PCR).

### Vaccinating calves younger than 12 months

By the time of our field visit, Rwanda has been vaccinating against FMD using FOTIVAX™ from Kenya (KEVEVAPI) once a year. It is a trivalent vaccine containing serotypes O, SAT1 and SAT2 with a recommendation of vaccinating twice or thrice a year for better protection [23]. We found that 57/142 (40.15%) of farmers did not vaccinate calves which are younger than 12 months of age. Our results also showed an association between farmers reporting not to vaccinate calves under 12 months and FMD outbreaks in their herds (OR = 0.707, *p* = 0.046, *n* = 137, CI = 95%) (Table 1).

**Table 1:** Multivariable model for risk factors for the occurrence of FMD outbreaks in herds raised in Eastern Rwanda

Parameter description	Estimate	Standard error	Significance	Odds ratio
Intercept	1.964	0.1336	0	7.13
Vaccinate calves under 12 months	− 0.347	0.1738	0.046	0.707
Not vaccinating calves under 12 months	0			

### Presence of small ruminants

**Fig. 1:**
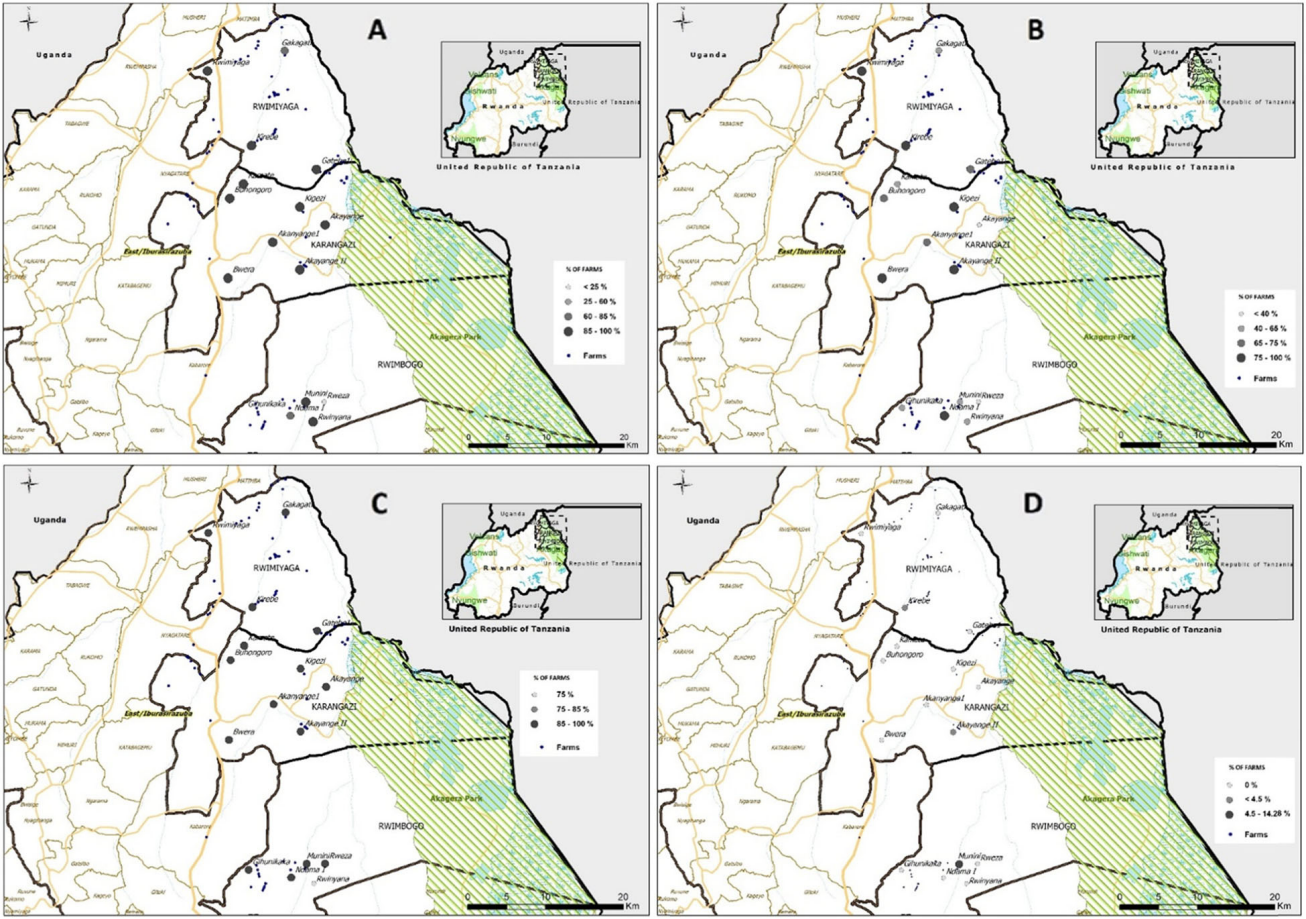
**a**: Map showing the proportion of farms where small ruminants are kept together with cattle per village (map created using QGIS v. 2.18.22 (https://qgis.org/downloads/)). **b**: Map showing the proportion of farms practising semi zero-grazing and free-ranging in each village (map created using QGIS v. 2.18.22 (https://qgis.org/downloads/)). **c**: Map showing the proportion of how farms are adjacent to each other in each village (map created using QGIS v. 2.18.22 (https://qgis.org/downloads/)). **d**: Map showing proportion of the non-fenced farms in each village (map created using QGIS v. 2.18.22 (https://qgis.org/downloads/))

In our study, small ruminants such as goats and sheep being kept together with cattle were reported in all villages as shown in the map below (Fig. 1a). Among the 143 respondents, only 13/143 (9.09%) kept cattle only while 129/143 (90.21%) reported mixed farms and one respondent did not answer to this question. Analyses showed that there are more chances (OR = 1.501, *p* = 0.163, *n* = 142 and CI = 95%) for FMD outbreaks to occur when there are sheep and goats on the same farm (Table 2).

**Table 2:** Univariable model for risk factors for the occurrence of FMD outbreaks in herds raised in Eastern Rwanda

Parameter description	Estimate	Standard error	Significance	Odds ratio
Natural breeding	0.486	0.3876	0.21	1.626
Artificial insemination	0			
Vaccinate calves under 12 months	− 0.347	0.1738	0.046	0.707
Not vaccinating calves under 12 months	0			
Presence of small ruminants	0.406	0.291	0.163	1.501
Absence of small ruminants	0			
Farm adjacent to another	−0.257	0.583	0.659	0.773
Farm isolated	0			
Not zero-grazing	−0.204	0.186	0.274	0.816
Zero-grazing	0			
Farm fenced	−0.255	0.712	0.720	0.775
Farm not fenced	0			
Farm adjacent to the park	−1.255	0.571	0.028	0.285
Farm not adjacent to the park	0			

### Breeding methods

We evaluated the impact of the breeding systems on the spread of the disease. We found that only 7 farmers out of 143 do use Artificial Insemination (AI) as a breeding method. Furthermore, the use of natural breeding methods increased the odds of FMD outbreak (OR = 1.626, CI = 95%) as compared to farms which were using AI as a breeding method (*p =* 0.21, *n* = 143) (Table 2). Due to the low number of farmers using AI, these results might be inconclusive and further analyses are necessary.

### Seasonality of FMD outbreaks and farming system

In this study, we also determined the influence of seasonality on outbreaks of FMD. A majority (85.31% with *p* < 0.01, *n* = 143) of the farmers interviewed reported that FMD is more likely to occur during the major dry seasons (the major one from June to September and a less severe one from December to February) than in the wet season. These months coincide to what was reported by Kerfua et al. [16] and the OIE records [14, 24]. We found that the majority (102/143 [71.33%]) (*p* < 0.01) of the farmers practising semi-zero grazing system and fewer (40/143[27.97%] practising strict zero grazing. No farmer responded to practice free-ranging system and one of the respondents did not provide an answer. Strict zero-grazing was practised in three villages only. In other villages, the majority of cattle move daily in search of water and animals from different farms are more likely to congregate at watering points. Rwimiyaga, Kirebe, Kigezi, Bwera and Akanyange II villages located in Nyagatare district and Ndama village located in Gatsibo district have more than 75% of the farmers practicing semi-zero grazing (Fig. 1b). Not practicing zero grazing system does not significantly increase the outbreak occurrence, and cannot be considered as a risk factor in this case (OR = 0.816, *p* = 0.274, CI = 95%) (Table 2). In addition, farmers practicing zero grazing reported higher incidence of outbreaks than those practicing semi-zero grazing system (Table 3).

**Table 3:** The proportion of farms for each farming systems practised in Nyagatare and Gatsibo districts of Eastern Rwanda and the impact on FMD outbreaks

	Farming system	
	Zero-grazing	Semi zero-grazing
	Number of farms (%)	
At least one outbreak in the last 5 years		
Yes	16/40 (40)	20/102 (19.6)
No	24/40 (60)	82/102 (80.4)
Total	40 (27.97)	102 (71.33)

### Proximity of farms to each other

We found that majority of the farms (140/143 [97.9%]) were adjacent to at least another farm (Fig. 1c), a situation that can lead to increased transmission of FMD in case there is an outbreak in one of the farms. Some (3/143 [2.1%]) of the visited farms were not fenced. None of the three farms that were not adjacent to another farm reported having had an FMD outbreak in the previous 5 years. When tested against the criterion of either having had at least one FMD outbreak in the farm the results were not significantly exploitable (OR = 0.773, *p* = 0.659 with *n* = 143 and CI = 95%).

### Wildlife-livestock interface as a risk factor

**Fig. 2:**
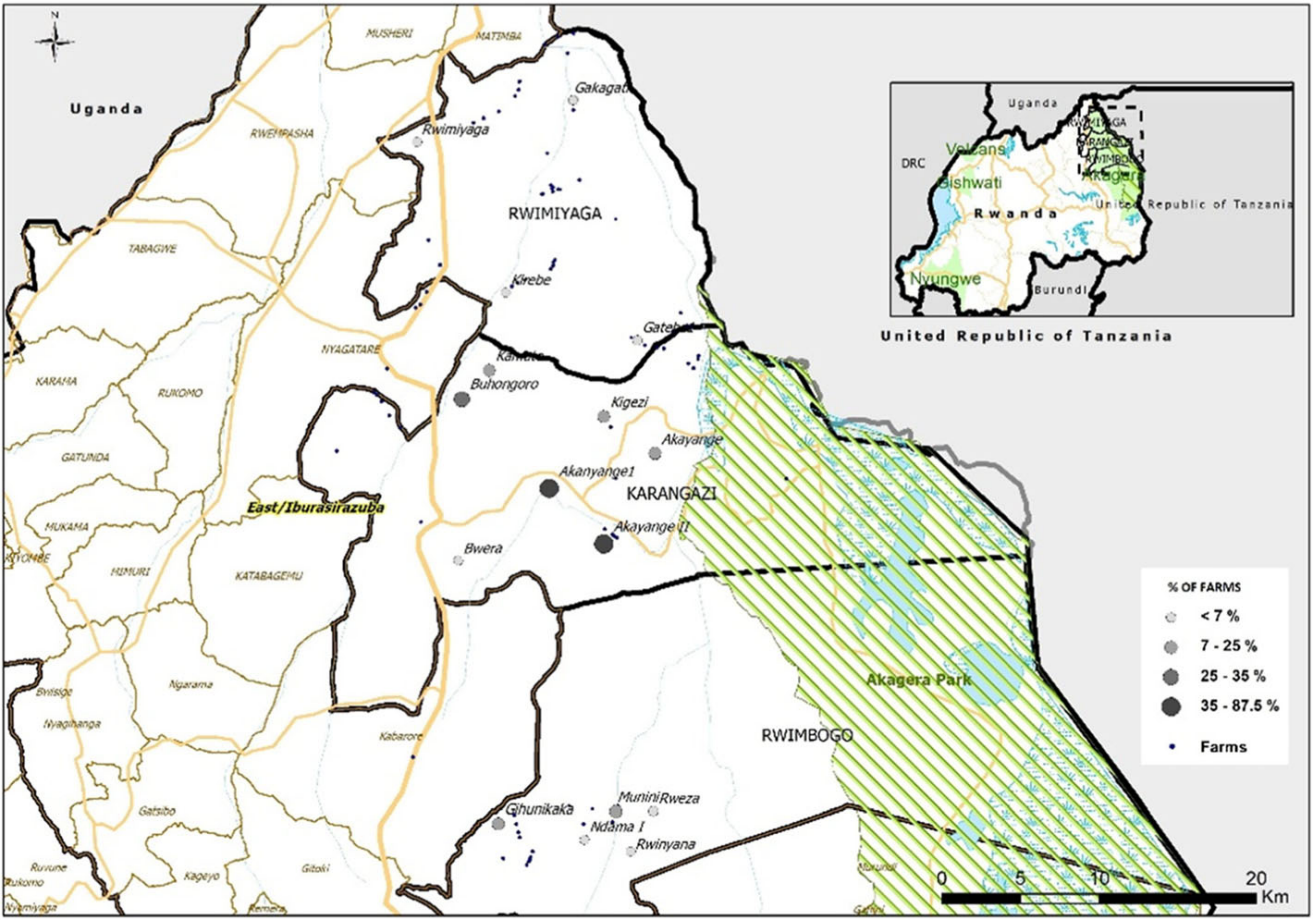
Map showing the proportion of farms at the interface with ANP per village in Eastern Rwanda (map created using QGIS v. 2.18.22 (https://qgis.org/downloads/))

Of the 27 farms located in Akanyange II village of Nyagatare district, 88.9% of them were adjacent to the ANP, indicating that domestic animals from this village had the highest chances of interacting with wildlife (Fig. 2). Overall, 36/143 farms were adjacent to ANP. Among these, 11.1% (4/36) reported having experienced FMD outbreaks in the previous 5 years against the remaining 88.9% (32/36) who reported not to have had FMD in their farms in that period. The other group of farms (105/143), 30.5% (32/143) reported having had FMD outbreaks while 69.5% (73/105) have not had FMD outbreaks in their farms in that period (OR = 0.285, *p* = 0.028, CI = 95%).

### Maps of selected risk factors

We mapped the risk factors such as farms that kept cattle, sheep and goats together (mixed farms), farming systems, non-fenced farms, closeness of various farms to each other and farms adjacent to the ANP. As shown in Fig. 1a, most of the farms in the villages had a mixed-farming system (raising cattle, sheep and goats together) with 129 out of 143 (90.21%) mixed farms. Mixed farms are evenly distributed throughout the Nyagatare and Gatsibo districts except for two villages in Nyagatare district, which had less than 85% of mixed farms. Figure 1b shows the proportion of farms practising semi zero-grazing and free-ranging per village. Observation shows that villages near rivers would have more farms not practising strict zero-grazing (> 75%). This is the case for Rwimiyaga, Kirebe, Kigezi, Akayange II and Bwera villages of Nyagatare district and Ndama I village in Gatsibo district.

As the Fig. 1d demonstrates, the fencing of the farms is well practised with an exception of Munini (> 4.5%) village of Gatsibo district having a higher percentage of unfenced farms when compared to other villages. Rwinyana village of Gatsibo district is the only one among all the villages with fewer farms (< 75%) that are not close to each other (Fig. 1c). Akayange I and Akayange II villages in Nyagatare district are the ones with a higher proportion of farms at the interface with ANP (Fig. 2). These two villages are also near a major road network linking ANP and other parts of the country. An outbreak in these two villages would easily spread to other parts due to its central position in Eastern Rwanda and the nearby road network.

## Discussion

### Vaccinating calves younger than 12 months

The trivalent (SAT1, SAT2 and O) Kenyan vaccine has been used to vaccinate cattle in Eastern Rwanda once a year. In the case of an FMD outbreak, a ring vaccination targeting cattle in the area has been practised. FMD vaccines are provided to farmers and subsidised by the government and this mitigates the risk of leaving behind some farms unvaccinated. Approximately 40% of the respondents were found not to vaccinate calves younger than 12 months during the vaccination programs. There may be a perception that calves are less impacted by FMD hence leading farmers to be reluctant in including them into vaccination programs [21]. A further study to understand what would have made a farmer not to include calves into vaccination programs is needed. Our data analysis revealed that failure to vaccinate calves younger than 12 months significantly increased the risks of FMD occurrence in the farms. This finding is consistent with previous studies in which vaccination of calves below 12 months have been found to enhance the protection of herds against FMD outbreaks [13, 25]. Hence, the ideal would be to clearly state with a data-backed decision at what age vaccination would not interfere with maternal antibodies and with a mature immune system to optimally respond to vaccination. Following the vaccine manufacturer’s instructions would have made this risk factor less confounding. Therefore, we recommend conducting more vaccinations a year as per the manufacturer’s instructions and regular vaccine matching studies. We also recommend a sensitization to farmers to include animals of all ages in vaccination and at least two vaccinations campaigns a year with booster doses.

### Mixed farms

Rearing of cattle together with sheep and goat in the same farm has been documented to be one of the risk factors responsible for FMD outbreak and transmission [26]. This may be a result of the fact that small ruminants show less severe clinical signs of FMD and are not usually included in vaccination programs [27, 28]. Balinda and colleagues (2009) reported a high prevalence of FMDV in small ruminants in Uganda and this has been linked to FMD outbreaks in cattle [29]. Our results flow in the same direction as the above findings in other places. Furthermore, in our study area, we have observed that small ruminants are reared separately from cattle, they tend to go more often and farther outside the farm for grazing and watering. However, the figures above lack a statistical significance to assert the above facts in this case. The low FMD rate may be because the 13/143 (9.09%) non-mixed farms were small-scale dairy farms, this may be the reason for the lower rate of FMD [21]. Therefore, considering the vaccination regime and the high number of small ruminants in Eastern Province, a deeper investigation on the role played by small ruminants could provide a basis for better control of FMD. From this, policy-makers would decide on whether to conduct more regular vaccination of only cattle with high-quality vaccines or if it is necessary to also include small ruminants in vaccination. Due to a cultural taboo for traditional cattle pastoralists to keep pigs, farmers in our study area reported not to keep pigs. We suggest further studies to focus on the role played by pigs in other regions of Rwanda.

### Breeding methods

There are reports that breeding methods using AI and natural breeding is responsible for the spread of FMD during outbreaks [30, 31]. However, testing and monitoring of bulls to provide pathogen-free semen could reduce the risk [32]. Indeed, we observed the AI centres used by the farmers usually screen the bulls for a range of animal infectious diseases including FMD; thereby reducing the risk of the disease transmission. However, further studies on the role of AI on the FMD transmission risk in Eastern Rwanda is still needed to confirm our results.

### Seasonality of FMD outbreaks and farming system

Most of the farmers interviewed reported the likeliness of more outbreaks during the dry season than in the wet season. Indeed, this finding is consistent with official records of the Rwanda Agriculture Board (Personal communication). In addition to OIE reports [14, 24], other studies have also reported more outbreaks of the disease during the dry season as opposed to the wet season [26, 33, 34]. Previous studies have documented that common watering point can provide a means for trans-farm transmission and spread of FMD [33, 35] and this is usually observed during prolonged droughts as reported in one study in Tanzania [36]. According to our observation, the trend in the area was to have a common valley dam where animals from different farms go to drink. The daily gathering of animals from different farms, as we observed during this study, was an important trend that can be responsible for disease spread. Other previous studies have also reported that herd contacts at watering points can be a risk for the introduction, spread and persistence of FMD [26, 35, 37, 38]. A previous study has established that uncontrolled cattle movements in East Africa are one of the risk factors responsible for the transmission and spread of FMD in the region [39]. To mitigate this problem, digging water dams or well within the farms seems to be an appropriate solution. Access to water on the farm is not always easy and affordable, especially to small-scale farmers. In India and Ethiopia, a subsidised system of solar-powered pumps has been adopted to provide water to the farms [40, 41].

### Proximity of farms to each other

FMD is usually spread by contact and to some extent by airborne means. Therefore, the transmission and spread of FMD during outbreaks tend to be faster in farms located close to each other [42–45]. Though many farms are adjacent to another farm in this study area, with a high *p* value, we did not find the results to be conclusive. Hence, we commend further investigations to look into this aspect. This may be because airborne transmission is much less when compared to direct contact transmission such as meeting at communal watering points. Previous studies have suggested that low humidity and high temperature could be responsible for reduced transmission of FMDV [46, 47]. Subsequently, it is possible that Eastern Rwanda may experience reduced FMD outbreaks when humidity is low and the temperature is high.

### Wildlife-livestock interface

Previous studies in Southern African countries have reported that wildlife, especially African buffaloes, are carriers of FMD virus indicating that the animals could be a source for the transmission of the virus [31, 48–52]. This often happens when cattle graze near the parks in the Southern African countries, especially during the dry season. In Southern African countries, the problem of wildlife-livestock interaction has been solved by fencing the national parks found in these countries to minimize contacts between wildlife and livestock [53]. Therefore, in order to reduce the human-wildlife-livestock interactions in Rwanda, the fencing approach has also been adopted particularly in Eastern Rwanda [54]. In this case, the Rwanda Development Board used an electric fence to separate the ANP from the livestock farmers. However, as we observed, some buffaloes were left outside the fenced park during the fencing programme. Nevertheless, there was not an immediate effect observed for farms adjacent to the ANP when compared to other farms in the study, probably park fencing mitigated this risk. Up to date, no laboratory-based results is pointing to the role played by buffaloes in Rwanda is available. A Pirbright research group suggested in 2018 that the role of African buffaloes in the transmission of FMD in East African might be different from what has been reported in Southern Africa countries. The latter study proposed a different control measure involving vaccination of cattle before an outbreak, as “a region-tailored” solution [55]. This proposal is supported by our findings, in which we surprisingly found that more farms have had FMD than the ones adjacent to the ANP fence. This can be linked to several factors such as that farms adjacent to the park are far from borders with less effect of transboundary animal movements. Moreover, ANP has been fenced since 2013 reducing the wildlife-livestock contact.

### Maps of selected risk factors

The mapping of risk factors is an important tool for understanding the epidemiology of FMD. The mapping has been used for generating a spatio-temporal distribution of the risk factors as reported in different parts of the world [26, 56–58]. Generating thematic maps of risk factors has also been reported to be of paramount importance in modelling and zoning of the disease in some countries [20, 58]. There is no previous study done to map the risk factors in Eastern Rwanda.

Rwinyana village of Gatsibo district is the only one among all the villages with fewer farms (< 75%) that are not close to each other (Fig. 1c). Akayange I and Akayange II villages in Nyagatare district are the ones with a higher proportion of farms at the interface with ANP (Fig. 2). These two villages are also near a major road network linking ANP and other parts of the country. An outbreak in these two villages would easily spread to other parts due to its central position in Eastern Rwanda and the nearby road network. For instance, infected cattle crossing roads to the watering points would leave infectious material behind that can attach to the tire surfaces of passing by vehicles. There is a need to confirm if farmers living near major roads move their animals more often than the farmers living far from major roads. In Nyagatare district, only 6% of the farmers have watering-points on their farms [59]. We believe that the use of on-farm watering-points would reduce the dependence on communal water points and hence reduce the odds of FMD transmission between different herds as reported previously [60, 61].

## Conclusion

We conclude that vaccinating calves under 12 months would protect the herds from the incursion of FMD. Therefore, we recommend vaccination programs that target both older and younger cattle, particularly following manufacturers’ instructions. Besides, farmers are aware that dry seasons are riskier than rainy seasons as long as FMD outbreaks are concerned. The proximity of farms to ANP or other farms including mixed farms that rear both cattle and small ruminants appear not to be statistically significant as risk factors. Further studies on the incursion of FMD in the area should focus on the role played by the domestic-wildlife interaction, the closeness of adjacent farms, the breeding system and awareness of farmers.

## Methods

### Study area

Nyagatare and Gatsibo districts experience low rainfall amounts with fewer rivers and are home to ANP, which has a considerable number of domestic and wildlife animals including African buffaloes (*Syncerus caffer*). The study area falls in the triangle neighbouring three countries namely, Tanzania, Rwanda and Uganda indicating risks for uncontrolled transboundary movements of animals between these countries. Since 1994, almost all of the reported FMD outbreaks (1996–1997, 2000–2001, 2004, 2005, 2006, 2013, 2015 and 2017) in the country have happened in Eastern Rwanda. Besides, in all these cases, biological samples were collected and taken to the laboratory to confirm the outbreak and detect the causative serotype(s) in laboratories.

### Study design

We designed a questionnaire [see Additional file 1] based on previously published papers on risk factors. Also, the Food and Agriculture Organization’s European Commission for the Control of FMD online document on FMD Investigation was used and is available at https://eufmdlearning.works/ [62]. The questionnaire covered several risk factors including farms not vaccinating calves less than 12 months of age, mixed farms keeping small and large ruminants [61], breeding system, seasonality of FMD outbreaks and farming systems [35, 63], farm adjacent to each other [64] as well as the wildlife-livestock interface [35]. In this study, zero-grazing stands for the system where livestock are reared inside the farm and water is available within the farm boundaries. Semi-zero grazing is for farmers who feed their animals within their farms’ boundaries but move their animals to communal watering points. Lastly, free-ranging is for farmers who graze and water their animals outside their farms. Most farmers in the area cultivate fodder mainly Napier grass (93.2%) [65].

### Target population and questionnaire administration

This study applied a cross-sectional study design and data were collected from Nyagatare and Gatsibo villages based on their proximity to ANP. A questionnaire was administered to all cattle farmers within the selected area covering 20 km distance from the electric fence of the ANP. This area covered the wildlife livestock interface where domestic and wildlife animals, especially African buffaloes, are likely to interact. We conducted interviews between May 2018 and August 2018. The information collected was documental on a paper-based questionnaire translated in Kinyarwanda and later entered in a spreadsheet of the Statistical Product and Service Solutions (SPSS Inc., IL, USA) version 16.0. The questionnaire had both open-ended and closed questions and was pre-tested on a smaller number of respondents to check for the clarity of the questions. Before each interview, respondents gave their verbal consent to proceed with the interview, after being briefed on the objectives and the expected outcomes of the study. The geographical coordinates of the farms were recorded using a Smartphone Application (Global Positioning System Coordinates Finder® by EzgApps). Using the geographical coordinates, maps were created in QGIS v. 2.18.22 (Las Palmas, USA).

### Data analysis

Data were coded in SPSS (SPSS Inc., IL, USA) for analysis, the codes were extensively revised to make sure that they are relevant based on the responses given by the farmers during the questionnaire administration. The descriptive statistics including proportion, means and categories of risk factors were generated. Data were also summarized using graphs. Inferential statistics such as chi-square (χ2) test was used to analyse the variables using the SPSS software (SPSS Inc., IL, USA). Where applicable, univariable and multivariable analyses were performed to estimate odds ratios (OR), using a criterion of whether a farm had experienced FMD outbreak in the last 5 years with a confidence interval of 95%.

## Supplementary information

**Additional file 1.** Questionnaire form for cattle farmers in Eastern Rwanda: this is the English version of the paper-based questionnaire that we developed and used to collect information for this study.

## Data Availability

The dataset backing the findings is available on https://figshare.com/articles/dataset/FMD_Risk_Factor_consolidated_data/12756134

## Abbreviations

FMD: Foot-and-Mouth Disease
FMDV: Foot-and-Mouth Disease Virus
QGIS: Quantum Geographic Information System
OR: Odd Ratio
SAT: Southern African Territories
PCP-FMD: Progressive Control Pathway for Foot-and-Mouth Disease
ANP: Akagera National Park
SPSS: Statistical Product and Service Solutions
AI: Artificial Insemination
CI: Confidence Interval
