# Supplementary material for: Risk factors for the incursion, spread and persistence of the foot and mouth disease virus in Eastern Rwanda
Source: BMC Vet Res. 2020 Oct 12;16:387. doi: 10.1186/s12917-020-02610-1 (PMC7552508; doi:10.1186/s12917-020-02610-1)
Supplement: Supplementary file 1 — Additional file 1. Questionnaire form for cattle farmers in Eastern Rwanda: this is the English version of the paper-based questionnaire that we developed and used to collect information for this study. [file 12917_2020_2610_MOESM1_ESM.pdf]

QUESTIONNAIRE FORM FOR CATTLE FARMERS IN EASTERN RWANDA

This questionnaire is for identification of risk factors of Foot and Mouth Disease Virus outbreaks in Eastern Rwanda. The interview has no intention to exploit the obtained data in any purpose other than research.

Interviewer: **UDAHEMUKA Jean Claude**

Date: ……./……./2018

Form number: ……………

Farm geographical coordinates: ………………………………………………………

Farmer general information

1. Names: ………………………………   …………………………
2. Farm code ……………….
3. Sex: a. Male
   b. Female
4. Age: ……………. years old
5. Marital status:a. single   d. Divorced
   b. Married   e. Widowed
   c. Engaged
6. District……………………………....
7. Sector…………………………………
8. Cell …………………………………...
9. Village (Umudugudu) ……………………………...

10. Educational status:
    a) Never attended formal education
    b) Primary school
    c) Secondary
    d) University
    e) Other (specify)

11. How many people do you have in your family? ………………

12. What is your social class? ……………………..[1]

Animal general information

13. How many cows do you have? ……………...
14. Which breed do you keep:

    a) Local (Ankole)
    b) Exotic
    c) Cross
    d) More than one breed

15. How are your cows acquired?
    1. Donation
    2. Buying
    3. Inherited
    4. Given by an NGO
    5. Government
    6. Other (Specify)

16. How many cows did you purchase / acquire in the last five years?...

17. How many calves younger than 6 months are in the herd?........................

18. How many calves are aged between 6 and 12 months? ....

19. How many bulls do you have? ....

20. What size is your farm?: ha …………

21. What type of farming do you practice?
    a. Strict Zero grazing
    b. Zero grazing with cattle walking for water
    c. Free ranging

22. Is your farm adjacent to another farm?
    a. Yes
    b. No

23. Do you have an off-farm job? (Is farming your only source of income?)
    a. Yes
    b. No

24. If yes, how would you rate the part played by your farm activities with your overall income? If no, how do your farming activities contribute to the overall income?
    a. Animal farm activities generate more than half of the income
    b. Animal farm activities generate half of the income
    c. Animal farm activities generate less than half of the income

25. Do you or any of your close neighbours have any of these animals: goat, sheep, pigs?
    a. Yes
    b. No

26. If there are pigs in the neighbourhood, are they swill fed?
    a. Yes
    b. No

27. When was the last FMD outbreak (Year and season of year)?
    …………./………………

28. How long did that outbreak last (in months)?.........................

29. In case of an outbreak, are samples being taken by animal health specialists?
    a. Yes
    b. No

30. If they collect samples, after how many days do they come prior to an outbreak?....................

31. How often are you likely to face an outbreak of FMD?
    a. Several times a year

b. Once in a year
c. Once in two years
d. Once in more than two years

32. According to your experience, at what period of year is FMD outbreak likely to surface (rain or dry season)?

33. What control measures are taken in case of an outbreak?
    a. Nothing
    b. Vaccination
    c. Quarantine
    d. Pre-emptive culling
    e. Depopulate after an outbreak

34. Did you face one or more outbreaks in the last five years?

35. Have you been facing an FMD outbreak annually for the last five years?

36. At the time of the most recent outbreak, did FMD occur in the radius of up to 5km?

37. How far is your usual cattle market (in Km)?...........................

38. Is your farm adjacent to the park?
    a. Yes
    b. No

39. Is your herd being systematically vaccinated?
    a. Yes
    b. No

40. If yes, how often?
    a. Once a year
    b. Twice a year
    c. Other (specify)………………………………

41. If no, what is/are reason(s)?
    a. The authorities don't propose it
    b. You don't have enough means for it
    c. You don't trust the vaccines
    d. Other (specify)………………………………

42. Do you vaccinate your calves younger than 12 months?
    a. Yes
    b. No

43. Is the time elapsed from the last vaccination of adults >6 months?
    a. Yes
    b. No

44. What was the morbidity rate during the last outbreak?..............................

45. What was the mortality during the last outbreak?..

46. How do you breed your cows?
    a. Artificial insemination (AI)
    b. Natural methods
    c. A combination of the two alternatives

47. If you use the natural method, do you use your own bulls or you borrow a bull from your neighbours?

48. Is your farm fenced?
    a. Yes
    b. No

49. Is there any restriction to a stranger to access your farm?
    a. Yes
    b. No

50. Do you have disinfectant bath at the entrance of your farm?
    a. Yes
    b. No

51. Where do you sell the milk?
    a. The nearest Milk Collection Centre (MCC)
    b. The nearest diary
    c. Informal selling
    d. Exclusively consumed by family members

Notes:

1: Answers will be according to social classification levels (Ubudehe) by the Ministry of Local Affairs of the Republic of Rwanda.
